# Histamine *N*-methyltransferase regulates aggression and the sleep-wake cycle

**DOI:** 10.1038/s41598-017-16019-8

**Published:** 2017-11-21

**Authors:** Fumito Naganuma, Tadaho Nakamura, Takeo Yoshikawa, Tomomitsu Iida, Yamato Miura, Anikó Kárpáti, Takuro Matsuzawa, Atushi Yanai, Asuka Mogi, Takatoshi Mochizuki, Nobuyuki Okamura, Kazuhiko Yanai

**Affiliations:** 1 Department of Pharmacology, Tohoku University Graduate School of Medicine, 2-1 Seiryo-machi, Aoba-ku, Sendai 980-8575 Japan; 2 Division of Pharmacology, Faculty of Medicine, Tohoku Medical and Pharmaceutical University, 4-4-1 Komatsushima, Aoba-ku, Sendai 981-8558 Japan; 3 Academic Research and Industrial Collaboration Management Office of Kyusyu University, 3-8-34 Momochihama, Sawara-ku, Fukuoka 814-0001 Japan

## Abstract

Histamine is a neurotransmitter that regulates diverse physiological functions including the sleep-wake cycle. Recent studies have reported that histaminergic dysfunction in the brain is associated with neuropsychiatric disorders. Histamine *N*-methyltransferase (HNMT) is an enzyme expressed in the central nervous system that specifically metabolises histamine; yet, the exact physiological roles of HNMT are unknown. Accordingly, we phenotyped *Hnmt* knockout mice (KO) to determine the relevance of HNMT to various brain functions. First, we showed that HNMT deficiency enhanced brain histamine concentrations, confirming a role for HNMT in histamine inactivation. Next, we performed comprehensive behavioural testing and determined that KO mice exhibited high aggressive behaviours in the resident-intruder and aggressive biting behaviour tests. High aggression in KO mice was suppressed by treatment with zolantidine, a histamine H2 receptor (H2R) antagonist, indicating that abnormal H2R activation promoted aggression in KO mice. A sleep analysis revealed that KO mice exhibited prolonged bouts of awakening during the light (inactive) period and compensatory sleep during the dark (active) period. Abnormal sleep behaviour was suppressed by treatment with pyrilamine, a H1R antagonist, prior to light period, suggesting that excessive H1R activation led to the dysregulation of sleep-wake cycles in KO mice. These observations inform the physiological roles of HNMT.

## Introduction

Histamine is a neurotransmitter that regulates a variety of physiological functions including sleep-wake cycles, appetite, memory and the stress response^1–4^. Histaminergic dysfunction is implicated in multiple neuropsychiatric disorders; for example, histamine deficits have been reported in Alzheimer’s disease and narcolepsy^5,6^. The apparent physiological importance of the central histaminergic system has accelerated attempts to pharmacologically manipulate brain histamine concentrations for the treatment of neurological disorders^7^.

Neurotransmitter clearance is an important factor determining brain neurotransmitter concentrations. Specifically, metabolizing enzymes such as acetylcholine esterase (AChE; EC 3.1.1.7) and catechol-*O*-methyltransferase (COMT; EC 2.1.1.6) play central roles in the maintenance of normal neuronal activity and brain homeostasis. Donepezil, an AChE inhibitor, and entacapone, a COMT inhibitor, were developed and implemented for the treatment of Alzheimer’s disease and Parkinson’s disease, respectively^8,9^. Based on these clinical applications, it can be hypothesised that histamine-metabolizing enzymes may represent an additional therapeutic target for the treatment of neurological disorders associated with decreased histaminergic activity in the brain. Yet, the physiological roles of histamine-metabolizing enzymes remain unclear.

Diamine oxidase (EC 1.4.3.22) is a histaminase that is primarily expressed in periphery, with negligible expression in the central nervous system (CNS)^10^. Conversely, histamine *N*-methyltransferase (HNMT; EC 2.1.1.8) is another histamine metabolizing enzyme that is expressed in various organs including the CNS^11^. HNMT is localised in the cytosol and metabolises histamine to 1-methylhistamine after extracellular histamine uptake into the intracellular space^12,13^. Although previous studies using HNMT inhibitors *in vivo* have provided evidence for the physiological importance of HNMT, the use of HNMT inhibitors as a tool for studying the role of HNMT in brain function is limited by low specificity^14^ and poor blood-brain barrier permeability^15^. Recent clinical studies have suggested that HNMT polymorphisms and associated changes in enzyme activity mediate pathological aspects of Parkinson’s disease and multiple sclerosis^16–18^. In particular, the Thr105Ile loss-of-function (i.e., decreased enzymatic activity) polymorphism was reported to exert protective effects in schizophrenia and attention deficit hyperactivity disorder^19,20^. Therefore, a better understanding of the role of HNMT in brain function is critical for pathological research as well as therapeutic development. On this premise, we evaluated the phenotypic characteristics and behaviours of *Hnmt*-deficient mice (KO) in order to clarify the importance of HNMT for histamine clearance and brain function.

## Results

### KO validation and general observations

The absence of HNMT expression in KO mice was confirmed using RT-PCR (Fig. S1A) and western blotting (Fig. S1B). Enzymatic assays confirmed an absence of HNMT activity in KO brain tissue (Fig. S1C). Physically, KO mice were indistinguishable from wild type mice (WT). KO mice matured normally and both male and female mutants were fertile. There were no significant effects of *Hnmt* deficiency on body weight (Fig. S2A) or food intake (Fig. S2B).

### Brain histamine abundance

**Figure 1:**
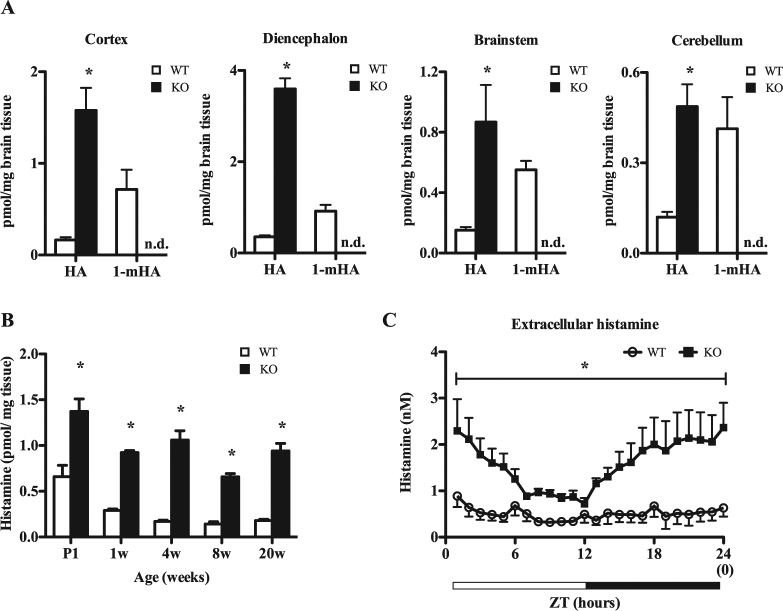
*Hnmt* deficiency increases brain histamine levels. (**A**) Histamine content in cortex, diencephalon, brainstem, and cerebellum homogenates (n = 5). 1-mHA, 1-methylhistamine; HA, histamine; n.d., not detected. (**B**) Histamine content in whole brain homogenates at various ages (n = 5–9). White bars, wild type (WT); black bars, knockout (KO). Student’s *t*-test, *p < 0.05. (**C**) Extracellular histamine concentrations in the hypothalamic area of WT (white circles) and KO mice (black square) for 24 h. Light period (white horizontal bar): ZT0-12, Dark period (black horizontal bar): ZT12-24 (n = 8).

We next examined the abundance of histamine in brain tissues and confirmed that histamine content was at least 5-fold higher in KO mice compared to WT mice in most brain regions (e.g., cortex, diencephalon, brainstem and cerebellum). Moreover, the primary product of HNMT activity (1-methylhistamine) was not detected in KO brain lysates (Fig. 1A). With regard to development, brain histamine concentrations were consistently increased in neonatal, adolescent and adult KO mice compared to age-matched WT mice (Fig. 1B), demonstrating a role for HNMT throughout development and adult life. Additionally, *in vivo* microdialysis revealed that KO mice had higher extracellular concentrations of histamine in the hypothalamic area, irrespective of light/dark cycles (Fig. 1C). Histamine was also elevated in some peripheral tissues of KO mice including the liver and kidney (Table S1). Conversely, HNMT deficiency did not affect brain dopamine, norepinephrine, serotonin or related metabolite concentrations in whole brain lysates (Table S2). These data indicated that HNMT deficiency had a broad impact on extracellular and intracellular histamine concentrations.

### Behavioural characterizations

**Figure 2:**
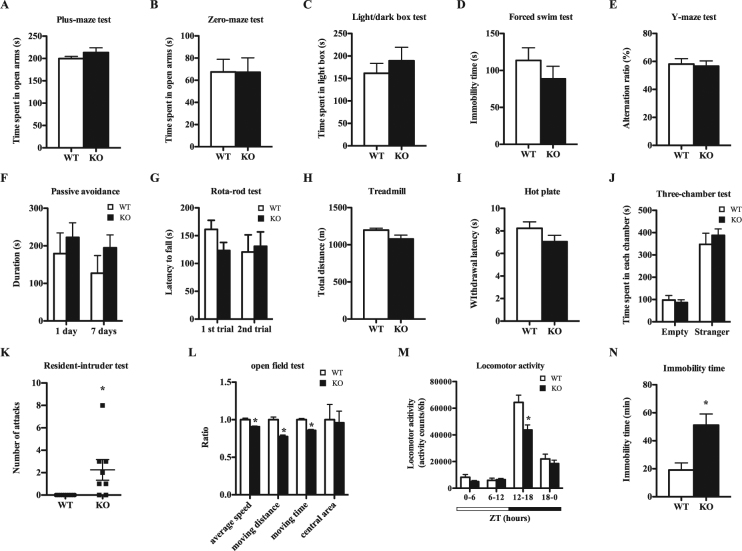
HNMT-deficient mice exhibit high aggressive behaviour and low locomotor activity. (**A**) Time spent in the open arms of the elevated plus-maze (n = 10–12). (**B**) Time spent in the open arms of the elevated zero-maze (n = 10–12). (**C**) Time spent in the light box in the light/dark box test (n = 5–8). (**D**) Immobility time in the forced swim test (n = 8). (**E**) Alternation ratio in the Y-maze test (n = 8). (**F**) Latencies at 1 day or 7 days after training in the passive avoidance test (n = 8). (**G**) Latency to fall in the first and second trials of the rotarod test. (**H**) Total distance in the treadmill test (n = 10). (**I**) Withdrawal latency in the hot plate test (n = 10–12). (**J**) Time spent in the empty and stranger chambers in the three-chamber test (n = 5–8). (**K**) The number of attacks in the resident-intruder test (n = 8–11). (**L**) Ratios of the average speed, distance, movement time, and central area duration in the open field test (n = 5–8) (Student’s *t-*test, *p < 0.05). The performance value of WT mice was set to 1. (**M**) Locomotor activities in the home-cage. Light period (white horizontal bar), ZT0-12; dark period (black horizontal bar), ZT12-24 (n = 10) (Student’s *t-*test, *p < 0.05). (**N**) Immobility time in the home-cage during the first 2 h of the dark period (ZT12-14; white bars, WT; black bars, KO) (n = 10) (Student’s *t-*test, *p < 0.05).

We next performed comprehensive behavioural testing to evaluate the behavioural effects of HNMT deficiency and elevated histamine in KO mice. KO mice did not exhibit anxiety-like behaviours in the elevated plus-maze (Fig. 2A), zero-maze test (Fig. 2B) or light/dark box test (Fig. 2C), or depression-like behaviour in the forced swim test (Fig. 2D) with respect to WT mice. Similarly, no deficits in working memory were identified in the Y-maze test (Fig. 2E) and learning/memory appeared normal in the passive avoidance test (Fig. 2F). Additionally, there were no significant differences between KO and WT mice in motor control (Fig. 2G), motor activity (Fig. 2H), pain sensitivity (Fig. 2I) or social interaction (Fig. 2J). However, KO mice showed significantly increased aggressive behaviours in the resident-intruder test compared to WT mice (Fig. 2K). Moreover, KO mice exhibited decreased locomotor activity in the open field test without changes in central area duration compared to WT mice (Fig. 2L). During home cage observation, KO mice exhibited decreased locomotor activity compared to WT mice during zeitgeber time (ZT) 12–18 (Fig. 2M), with the longest periods of immobility occurring during ZT12-14 (Fig. 2N).

### Aggression

**Figure 3:**
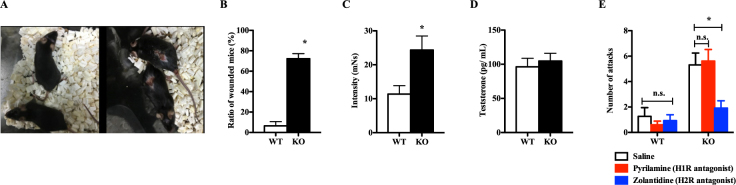
Increased aggression in *Hnmt*-deficient mice is driven by H2R activation. (**A**) Images of wild type (WT, left) and knockout (KO, right) mice in their home cages. Most KO mice had skin injures. (**B**) Ratios of wounded mice (n = 26–28). (**C**) Intensity of biting behaviours on the aggressive response meter (n = 8) (Student’s *t*-test, *p < 0.05). (**D**) Serum testosterone concentrations (pg/mL). (**E**) The number of attacks over 5 min in the resident-intruder test with histamine receptor antagonist pretreatment. White bar, saline; red bar, 10 mg/kg pyrilamine (H1R antagonist); blue bar, 10 mg/kg zolantidine (H2R antagonist) (two-way ANOVA with Bonferroni correction; *p < 0.05; n.s.: not significant).

To confirm that increased aggressive behaviours were related to elevated brain histamine in KO mice, we performed a more detailed analysis of the behavioural data. Male KO mice often attacked other males, such that >70% of male KO mice had skin wounds in their home cage (Fig. 3A and B). On this premise, we performed the aggressive biting behaviour (ABB) test to objectively evaluate aggressive behaviours and found that ABB intensity was significantly increased in KO mice compared to WT mice (Fig. 3C). Although previous studies have reported increased serum testosterone in high-aggression male mice^21^, serum testosterone levels were not significantly different between WT and KO mice (Fig. 3D), indicating that behavioural changes were likely attributable to elevated brain histamine. Next, we examined the histamine receptor(s) involved in promoting aggression in KO mice. Treatment with zolantidine, an H2R antagonist, significantly decreased the number of attacks by KO mice in the resident-intruder test, whereas pyrilamine, an H1R antagonist, had no effect (Fig. 3E). These results indicated that elevated histamine and H2R activation related to HNMT deficiency drove aggressive behaviours in KO mice.

### Sleep analysis

**Figure 4:**
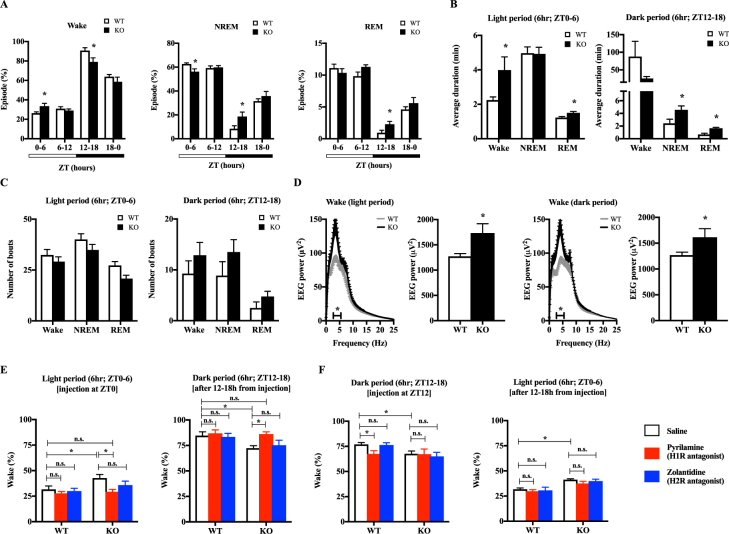
Sleep-wake cycle abnormalities in *Hnmt*-deficient mice are driven by H1R activation. (**A**) The ratios of wakefulness, NREM sleep, and REM sleep on the indicated Zeitgeber time (ZT) periods (n = 8). Light period (white horizontal bar), ZT0-12; dark period (black horizontal bar), ZT12-0 (n = 8, Student’s *t*-test; *p < 0.05). White bars, wild type (WT); black bars, knockout (KO). (**B**) Average duration of bouts during ZT0–6 and ZT12–18 (n = 8) (Student’s *t*-test, *p < 0.05). (**C**) Number of bouts during ZT0-6 and ZT12-18 (n = 8). (**D**) The spectral distribution of cortical EEG power density during wakefulness in the light and dark periods. Grey line, WT; black line, KO. Horizontal bars indicate statistical differences between the WT and KO groups. Bar graphs show the total power of EEG between 3.0 and 5.5 Hz. White bar, WT; black bar KO. (n = 8) (Student’s *t*-test, *p < 0.05). (**E**) Effects of pyrilamine and zolantidine administration at ZT0 on wakefulness in WT and KO mice during ZT0-6 and ZT12-18. (**F**) Effects of pyrilamine and zolantidine administration at ZT12 on wakefulness in WT and KO mice during ZT12-18 and ZT0-6. White bar, saline; red bar, 10 mg/kg pyrilamine (H1R antagonist); blue bar, 10 mg/kg zolantidine (H2R antagonist) (n = 6–8) (two-way ANOVA with Bonferroni multiple comparisons test; *p < 0.05; n.s.: not significant).

The observation that KO mice exhibited prolonged immobility during the dark period (Fig. 2N) led us to hypothesise that KO mice experienced sleep-wake cycle deregulation. We tested this hypothesis by performing a sleep analysis with electroencephalography (EEG) and electromyography (EMG) recordings. The sleep analysis revealed that KO mice exhibited increased wakefulness during ZT0-6 (inactive period) and decreased wakefulness during ZT12-18 (active period) compared to WT mice (Fig. 4A). Additionally, the average wake bout duration in KO mice was increased during ZT0-6 compared to WT mice (Fig. 4B), without a change in the total number of wake and sleep bouts (Fig. 4C). KO mice also exhibited increased EEG activity in the 3.0–5.5 Hz range during wakefulness compared to WT mice during both the light and dark periods (Fig. 4D). During NREM sleep, slow-wave EEG activity (0.5–4.0 Hz) was not significantly different between groups, but EEG theta activity (5.0–10.0 Hz) was lower in KO mice compared to WT mice during both the light and dark periods (Fig. S3A). EEG activities during REM sleep were not significantly different between groups (Fig. S3B).

Total daily amount of wakefulness/sleep is controlled in a homeostatic manner^22^ and our sleep analysis showed that wakefulness was increased during light period and decreased during dark period in KO mice. Thus, we hypothesized that elevated histamine in KO mice increased wakefulness during light period as a wake-promoting amine, resulting in the compensatory decrease of wakefulness during dark period. Pyrilamine treatment at ZT0 normalized the extended wakefulness during ZT0-6 in KO mice (Fig. 4E). Although pyrilamine is eliminated after 6 h from injection^23^, decreased wakefulness in KO mice during ZT12-18 was also cancelled. However, pyrilamine treatment at ZT12 did not affect abnormal wakefulness in KO mice (Fig. 4F). Zolantidine treatment had no effect on wakefulness in WT or KO mice (not statistically different). These data demonstrated that elevated histamine in KO mice directly increased wakefulness during light period through H1R signaling, and the decreased wakefulness during dark period in KO mice was compensatory response to the wake prolongation during light period.

## Discussion

In this study, we demonstrated that HNMT deficiency resulted in increased brain histamine, high aggressive behaviours and sleep-wake cycle abnormalities in mice.

Histamine in the nervous system was first identified approximately three decades ago^24^. Since then, numerous studies have revealed that histamine plays multiple roles in brain health and disease^25,26^. Decreased brain histamine causes neurobehavioural symptoms such as anxiety-like behaviours and impaired learning/memory^27,28^. In contrast, increased brain histamine produces appetite suppression, anxiolytic actions and improves cognitive function^29–31^. Several pharmaceutical companies have developed H3R antagonists/inverse agonists, which weakly (<2-fold) and transiently increase brain histamine levels^30^, for the treatment of obesity, Alzheimer’s disease and schizophrenia^32^. In the present study, HNMT deficiency increased brain histamine by >6-fold from the neonatal period; however, increased histamine in KO mice had no effect on food intake, anxiety-like behaviours or learning and memory. Similar discrepancies between the effects of neurotransmitter versus transporter manipulation have been observed with regard to other systems; in previous studies, serotonin transporter (Sert) inhibitors showed anxiolytic effects^33^, whereas Sert gene disruption did not affect anxiety-like behaviours in mice^34^. These differences might represent the difficulty of comparing pharmacological effects and genetic mouse models.

Numerous studies have demonstrated an association between elevated serum testosterone and increased aggressive behaviours^35^. Although Mondillo *et al*. previously reported the ability of histamine to induce testosterone synthesis and secretion in murine Leydig cells^36^, we did not observe any effect of HNMT deficiency on serum testosterone level. Thus, it can be hypothesised that elevated histamine in the CNS of KO mice directly produced aggressive behaviours. Previous studies have reported a role for the H2R in aggression in mice and rats^37,38^, supporting our findings. H2R is expressed in the thalamus, hypothalamus, basal ganglia and amygdala in guinea pigs^39^ and rats^40^, which represent important regions for emotion and aggression^41^. Thus, excessive H2R activation in these regions may have induced high aggression in KO mice. Future studies should clarify the exact brain regions responsible for H2R-mediated aggression in mice.

H1R antagonists produce sedative effects and are sometimes used as sleeping pills; accordingly, histamine is widely recognised as a wake-promoting amine^42,43^. Extended wakefulness in light period associated with HNMT deficiency was inhibited by pyrilamine, emphasizing a role for the H1R in wakefulness. Our sleep analysis using pyrilamine demonstrated that wake prolongation by H1R activation increased wakefulness during light period. Previous studies also showed that elevated histamine promoted the wakefulness only during light period^23,44^. Yet, it was still unknown why histamine elevation throughout day had a wake-promoting effect on light period but not dark period. In addition, we found that HNMT deficiency affected the duration of wake bouts but not the number of wake bouts. Previous study showed that the firing rates of tuberomammillary nucleus (TMN), a main source of histamine-producing neurons, increased after wake transition^45^. These data suggest that histamine signalling through H1R is important for not generation but maintenance of wakefulness. H1R is highly expressed in the cortex, hypothalamus and basal ganglia, which are important brain regions for sleep-wake homeostasis^46^. Additionally, various other sleep/wake-related neurotransmitters and neuropeptides have functional roles in these brain regions^47–49^. Thus, further studies are needed to identify interactions between the H1R histamine system and other neuronal systems in sleep-wake cycle regulation.

Our sleep analysis also revealed that activity in the high-delta/low theta frequency range was significantly increased in KO mice during wakefulness. Parmentier and colleagues reported a sleep analysis of histidine decarboxylase (HDC)-deficient mice and identified decreased EEG power around 5.0 Hz^50^. Additionally, Takahashi and colleagues reported that excitatory stimulation of the TMN induced EEG activity in a similar range^51^. These findings suggest that the firing rate induced by histamine receptor activation may be around 5.0 Hz, and that this could be a characteristic pattern of histaminergic output for arousal. Further, in humans, enhanced cortical slow wave activity is associated with aggressive behaviours^52^, indicating that abnormal EEG activity in KO mice may have also been related to the observation of high aggression.

This study has some limitations which have to be pointed out. We used a conventional knockout technology to produce abnormalities in the CNS histaminergic system. Thus, region-specific *Hnmt* deletion using a conditional knockout strategy should be used to clarify the brain areas responsible for the observed aggression and sleep-wake cycle deregulation in this study. Moreover, since KO mice were evaluated under normal conditions, additional studies employing stress conditions may better inform the involvement of HNMT in psychological and neuropsychiatric disease. Finally, future works should examine the importance of HNMT in rodent models of neurodegenerative diseases such as Alzheimer’s disease and Parkinson’s disease.

In conclusion, HNMT plays a crucial role in regulating brain concentrations of histamine, and accordingly may regulate aggression as well as the sleep-wake cycle. Future studies are required to confirm and extend our findings in other rodent models and eventually humans.

## Methods

### Animals

The care and use of animals in this study was conducted in accordance with the Principles for the Care and Use of Research Animals of Tohoku University, Sendai, Japan, and all animal as well as gene-recombination experiments were given ethical approval from the Tohoku University Centres for Laboratory Research and Tohoku University Centres for Gene Research, respectively.

Adult male inbred C57BL/6J mice (8–12 weeks old) were used in all experiments. Hnmt^tm1a (KOMP) Wtsi^ mice were purchased from the KOMP repository (University of California, Davis, CA, USA, project ID; CSD34462). Mice were maintained on a 12-h light-dark cycle (on the indicated Zeitgeber time (ZT); where ZT0 is light onset and ZT12 is light shutoff) in a room with regulated humidity and temperature and group housed in a maximum of five animals per cage with free access to food and water. All behavioural experiments were performed during the light period.

### Histamine and 1-methylhistamine measurements

Histamine and 1-methylhistamine were measured by HPLC systems as previously described^12^. Briefly, after perfusion with ice-cold PBS, mouse brains were harvested immediately. Each brain region was homogenised in a 6-times volume of 0.4 M perchloric acid. After repeated centrifugation, samples were applied to an HPLC system.

### *In vivo* microdialysis

Extracellular histamine was collected using *in vivo* microdialysis system as previously described^4^. Briefly, a guide cannula (EICOM, Kyoto, Japan) was implanted stereotactically into the hypothalamus (AP: −1.5 mm, ML: +0.5 mm, DV: −3.5 mm from bregma). After 1 week from surgery, a 2-mm membrane length of the microdialysis probe (EICOM) was inserted into the guide cannula and used to perfuse artificial CSF solution. Dialysate was collected every 30 min and applied to the HPLC system.

### Elevated plus-maze test

The elevated plus-maze test was performed as previously described^53^. Briefly, mice were allowed to move freely in the maze (EPM-04^®^; Muromachi, Tokyo, Japan) for 10 min. Time spent in the open arm was measured as a parameter of anxiety-like behaviour using an overhead camera and tracking system (SMART^®^; Panlab, Barcelona, Spain).

### Elevated zero-maze test

The elevated zero-maze test was performed as previously described^4^. Briefly, mice were allowed to move freely in the maze for 5 min. Time spent in the open area was measured as a parameter of anxiety-like behaviour.

### Light/dark box test

The light/dark box test was performed using a box divided into two compartments by a partition with a small aperture as previously described^27^. Briefly, time spent in the dark room was measured as a surrogate for anxiety-like behaviour. Each mouse was placed in the light box and allowed to move freely for 10 min. Tracking equipment and software (Opto-Max^®^; Columbus Instrument, Columbus, OH, USA) were used to detect each mouse’s position and movement.

### Forced swim test

The forced swim test was performed as previously described^54^. Briefly, a 2-L transparent glass beaker filled with water to 15 cm from the bottom was used as the pool. Mice were placed in the beaker and allowed to swim undisturbed for 6 min. Immobility time during the 6-min period was measured as a surrogate of depression-like behaviour.

### Y-maze spontaneous continuous test

Working memory was examined by measuring the spontaneous continuous alternation ratio in the Y-maze test as previously described^27^. Briefly, each mouse was allowed to explore the Y-maze for 8 min. An overhead camera and tracking system (SMART^®^; Panlab) were used to record and measure mouse behaviours.

### Passive avoidance test

The passive avoidance test was performed as previously described (PA-M, O’hara & CO, Tokyo, Japan)^27^. Briefly, during training sessions, each mouse was placed into the light compartment. When the mouse stepped into the dark compartment, a continuous current foot shock (0.5 mA, 5 s) was delivered. At 1 and 7 days after training, a test trial was conducted and the latency to step into the dark compartment was recorded with a cut-off time of 5 min.

### Rotarod test

The rotarod test was performed as previously described^55^. Briefly, the apparatus was a single-lane rotarod (MK-630^®^; Muromachi) that turned with an initial speed of 4 rpm and gradually accelerated at a rate of 0.2 rpm/sec. Latency to fall from the rod was measured with a cut-off time of 3 min.

### Treadmill test

Motor ability was analysed using a treadmill as previously described^56^. This experiment was performed using a mouse treadmill (MK680^®^; Muromachi). Total running distance was calculated for each mouse with cutoff distance of 1355 m (final speed, 20 m/min).

### Hot plate test

The hot plate test is widely used to investigate nociception and pain-like responses in mice^57^. The temperature of the hot plate surface (DS-37, Ugo Basile, Varese, Italy) was maintained at 55 °C and withdrawal or licking of the paw was considered to be a pain-like response. Latency to the display of a pain-like response was measured.

### Three-chamber sociability test

Social interaction was examined using the three-chamber sociability test as previously described^27^. Briefly, after a 10-min habituation period, an empty transparent cup (empty) and a transparent cup containing an unfamiliar mouse (stranger) were introduced into separate chambers. Each mouse was allowed to explore for 10 additional min. An overhead camera and tracking system (SMART^®^; Panlab) were used to record and measure the latency to enter each chamber.

### Resident-intruder test

The resident-intruder test was performed as previously described^58^. Briefly, male mice (resident) were housed individually for three days before the testing day to increase territorial motivation. Then, an unfamiliar male mouse (intruder, C57BL6 WT) was introduced into the resident cage. The two mice were allowed to interact freely for 5 min and the number of attacks during this period were counted. Histamine receptor antagonists or saline were injected intraperitoneally 1 h prior to experiments.

### Open field test

Open field test was performed as previously described^27^. Briefly, each mouse was placed into the centres of a square area (Muromachi) and allowed to explore freely for 30 min. The total travel distance, average speed, movement time, and time spent in the central area were tracked using a photo-beam apparatus (BTA-1^®^, Muromachi).

### Home cage activity test

The home cage activity was performed as previously described^27^. Briefly, mice were transferred to individual home cages and habituated for 3 days. Thereafter, the locomotor activity of a mouse was measured by an activity-monitoring system (SUPERMEX, Muromachi) with infrared-beam apparatus and digitally converted it to activity counts by CompACT AMS^®^ (Muromachi). The activity count was calculated every 5 min epoch and recorded continuously for 24 h. Total immobility time was calculated from the number of epochs without any activity counts.

### Aggressive biting behaviour (ABB) test

ABB was measured using an aggression response meter (ARM-001^®^, Muromachi) as previously described^59^. Briefly, before ABB measurement, the stick was used to touch the abdomen of the mouse 30 times in order to provoke/irritate the mouse. In practical ABB measurements, the sticks approached the mouse’s face without touching it. When the mouse bit the stick, the load sensor detected the dynamic strength and duration of biting behaviour. Measurements were performed 30 times (10-s intervals) in the practical session, and the average intensity and number of responses were recorded.

### Testosterone measurement

Serum testosterone concentration was measured using a testosterone EIA kit (Cayman, Ann Arbor, MI, USA).

### Sleep analysis

Mouse EEG/EMG were measured as previously described^60^. Mice were implanted with a head mount for measuring EEG and EMG (Pinnacle Technology, Laurence, KS, USA). One week after surgery, EEG/EMG signals were acquired by using 3-channel EEG/EMG tethered system (Pinnacles Technology) and digitalized using SIRENIA SLEEP PRO^®^ software (Pinnacles Technology). Corrected EEG/EMG data were analysed using Sleep Sign^®^ Software (Kissei Comtec, Matsumoto, Japan). Histamine antagonists or saline were injected intraperitoneally just prior to ZT0 or ZT12.

### Statistical Analysis

Comparisons of tissue histamine content and behavioural data between WT and KO mice were generally performed using two-tailed paired Student’s *t-*tests. Behavioural study data with pharmacological treatment were analysed using two-way ANOVAs with the Bonferroni correction using GraphPad Prism^®^ version 5.0 (GraphPad, La Jolla, CA, USA). All data are presented as the mean ± standard error. Differences were considered to be statistically significant when P was less than 0.05.

## Electronic supplementary material

Supplementary information
