# Supplementary material for: Histamine N-methyltransferase regulates aggression and the sleep-wake cycle
Source: Sci Rep. 2017 Nov 21;7:15899. doi: 10.1038/s41598-017-16019-8 (PMC5698467; doi:10.1038/s41598-017-16019-8)
Supplement: Supplementary file 1 — Supplementary information [file 41598_2017_16019_MOESM1_ESM.pdf]

Supplementary Information

Histamine *N*-methyltransferase regulates aggression and the sleep-wake cycle

Fumito Naganuma[a, b], Tadaho Nakamura[a, b], Takeo Yoshikawa[a] *, Tomomitsu Iida[a], Yamato Miura[a], Anikó Kárpáti[a], Takuro Matsuzawa[a], Atsushi Yanai[a], Asuka Mogi[a], Takatoshi Mochizuki[c], Nobuyuki Okamura[a, b] and Kazuhiko Yanai[a].

[a] Department of Pharmacology, Tohoku University Graduate School of Medicine, 2-1 Seiryo-machi, Aoba-ku, Sendai, 980-8575 Japan

[b] Division of Pharmacology, Faculty of Medicine, Tohoku Medical and Pharmaceutical University, 4-4-1 Komatsushima, Aoba-ku, Sendai, 981-8558 Japan

[c] Academic Research and Industrial Collaboration Management Office of Kyusyu University, 3-8-34 Momochihama, Sawara-ku, Fukuoka, 814-0001 Japan

***Corresponding author:** Takeo Yoshikawa, M.D., Ph.D.

Department of Pharmacology, Tohoku University Graduate School of Medicine, 2-1 Seiryo-machi, Aoba-ku, Sendai, 980-8575 Japan. Phone: +81-22-717-8058, Fax: +81-22-717-8060, Email: tyoshikawa@med.tohoku.ac.jp

Supplementary figure 1

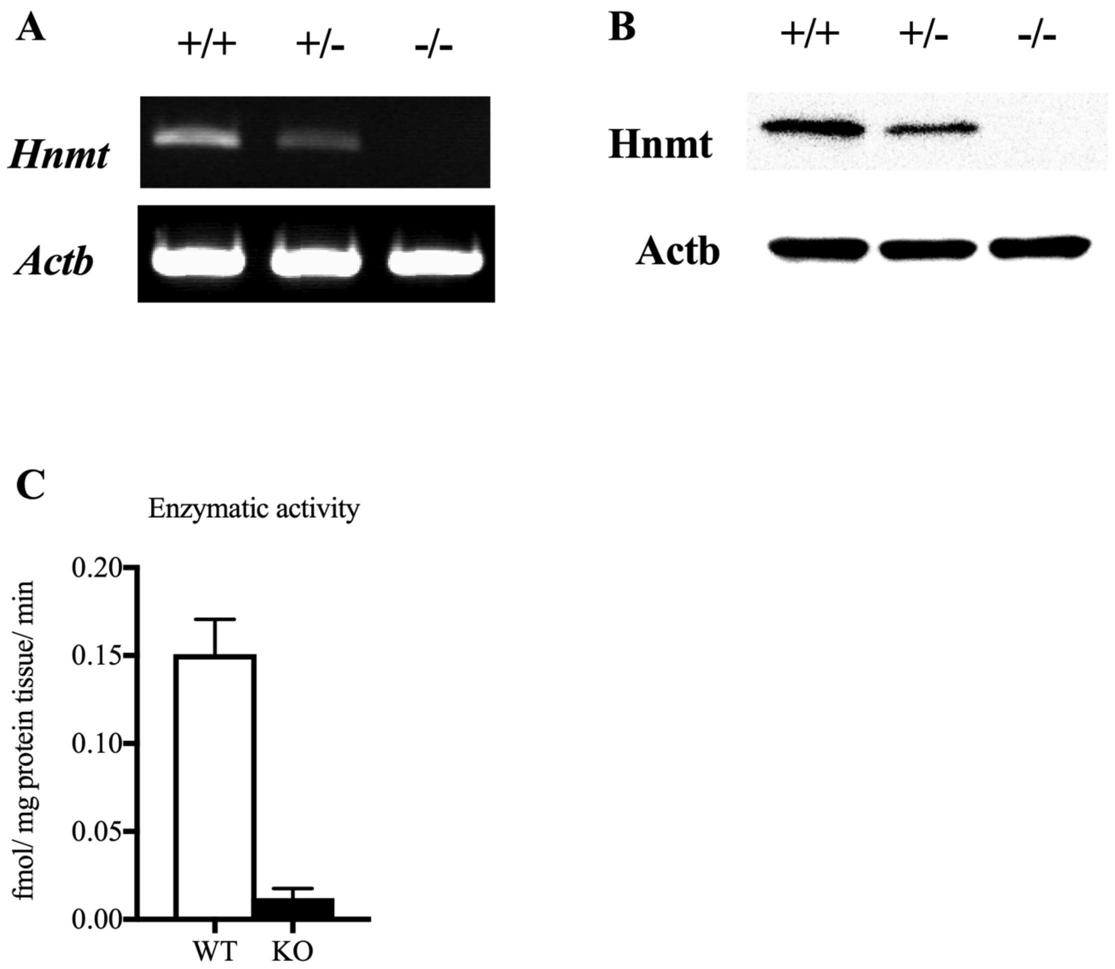

Fig. S1. HNMT expression and enzymatic activity.

The HNMT deficiency was confirmed by RT-PCR (A) and western blot (B). The expression of HNMT (Upper) and ACTB (β-actin) (lower, as a positive control). +/+, wild type mouse; +/-, heterozygous mouse; -/-, homozygous mouse. (C) Enzymatic activity of HNMT (n = 5).

Supplementary figure 2

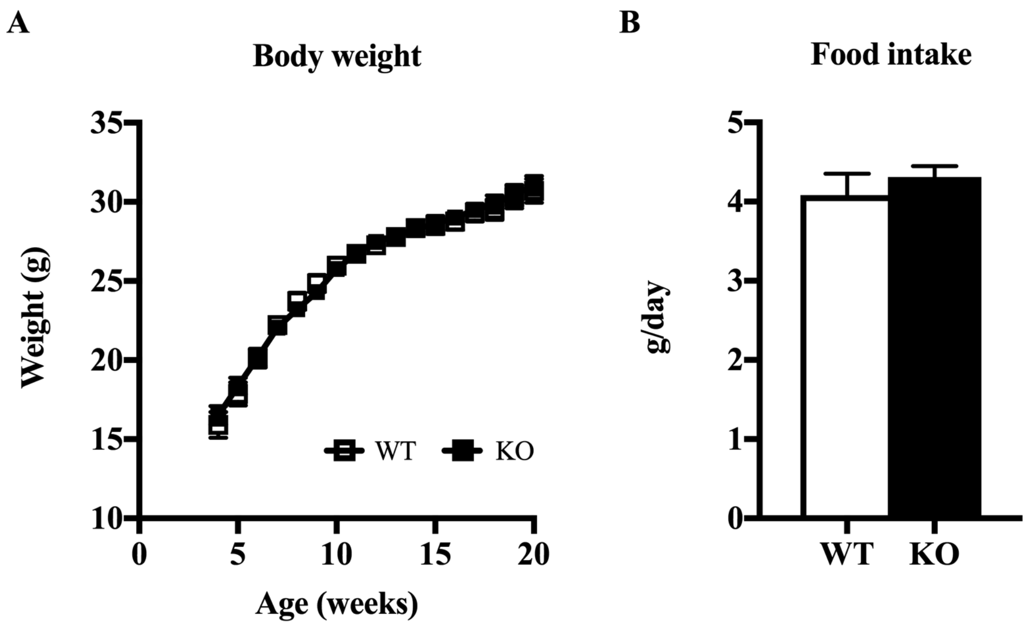

Fig. S2. Body weight and the amount of food intake.

(A) Body weight of WT (white circles) and KO (black circles) for 20 weeks (n = 15-18).

(B) The amount of food intake of WT (white circles) and KO (black circles) for 24 hours (n = 10).

Supplementary Figure 3

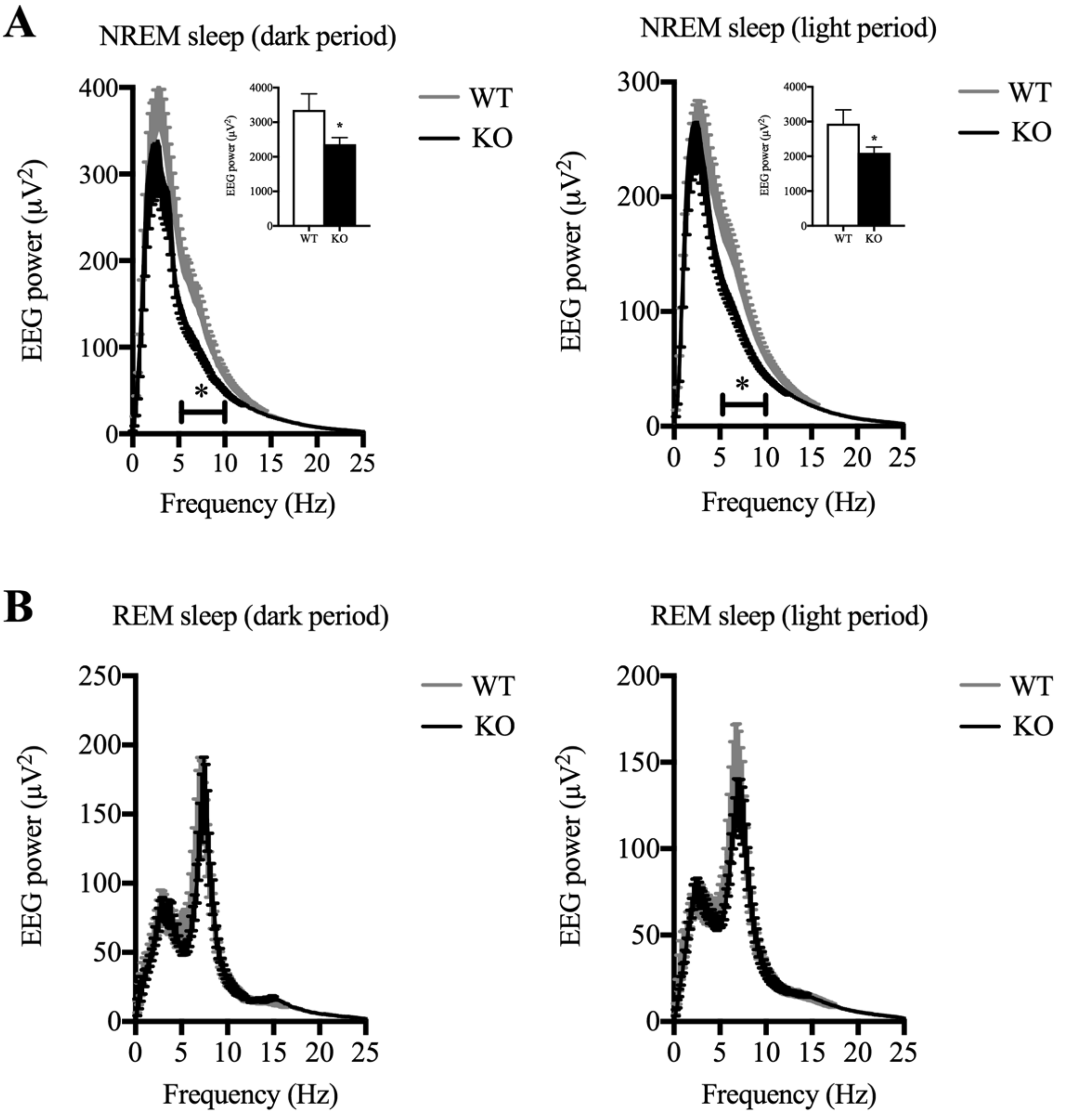

Fig. S3. Spectral distribution of cortical EEG power density during NREM sleep and REM sleep

(A) The spectral distribution of cortical EEG power density during NREM sleep in dark period and in light periods. Horizontal bars indicate statistical analysis between WT and

KO mice. Bar graphs showed the total power of EEG between 5.0 and 10.0 Hz. White bar, WT; black bar, KO (n = 8). (Student's *t*-test, * $p < 0.05$). (B) The spectral distribution of cortical EEG power density during REM in dark period and in light periods (n = 8).

Supplementary Table 1

Histamine concentrations in the homogenate of brain and peripheral tissues.

		WT (pmol/ mg tissue)	KO (pmol/ mg tissue)
Brain	HA	0.23 ± 0.026	1.2 ± 0.13 *
	1-mHA	0.23 ± 0.016	n.d.
Serum	HA	0.042 ± 0.0090	0.22 ± 0.042 *
	1-mHA	0.025 ± 0.0059	n.d.
Heart	HA	0.61 ± 0.028	1.04 ± 0.13 *
	1-mHA	0.026 ± 0.010	n.d.
Lung	HA	0.53 ± 0.0095	0.64 ± 0.14
	1-mHA	0.082 ± 0.040	n.d.
Stomach	HA	3.7 ± 0.51	4.5 ± 1.1
	1-mHA	0.2 ± 0.053	n.d.
Liver	HA	0.26 ± 0.034	0.67 ± 0.064 *
	1-mHA	0.19 ± 0.025	0.39 ± 0.12
Spleen	HA	2.6 ± 0.034	8.4 ± 0.58 *
	1-mHA	0.091 ± 0.014	n.d.
Kidney	HA	0.24 ± 0.057	0.57 ± 0.096 *
	1-mHA	0.12 ± 0.028	n.d.
Skin	HA	72 ± 14	77 ± 6.1
	1-mHA	n.d.	n.d.

HA, histamine; 1-mHA, 1-methylhistamine; n.d., not detected. (n = 5) (Student *t*-test, * $p < 0.05$).

Supplementary Table 2

Monoamine neurotransmitters and their metabolites concentrations in the whole brain lysate.

	WT (pmol/mg tissue)	KO (pmol/mg tissue)
5-HT	3.5 ± 0.26	3.9 ± 0.23
DA	9.0 ± 0.73	9.3 ± 0.34
NE	7.1 ± 0.47	7.6 ± 0.28
5-HIAA	1.2 ± 0.037	1.2 ± 0.032
DOPAC	0.87 ± 0.082	0.78 ± 0.048
3-MT	2.2 ± 0.11	2.4 ± 0.12
HVA	0.97 ± 0.20	0.76 ± 0.071

5-HT, serotonin; DA, dopamine; NE, norepinephrine; 5-HIAA, 5-hydroxyindole acetic acid; DOPAC, 3,4-dihydroxyphenyl acetic acid; 3-MT, 3-methoxythyramine; HVA, homovanillic acid (n = 5).

Supplementary Methods

RT-PCR

The NucleoSpin® RNA (MACHEREY-NAGEL, Duren, Germany) was used to isolate total RNA from brain tissues. A PrimeScript® II first-strand cDNA synthesis kit (Takara, Otsu, Japan) was used to reverse transcribe total RNA (500 ng) with oligo-dT and random hexamers. Diluted reverse-transcribed samples (equivalent to 500 pg of total RNA) were amplified by a DNA polymerase (Takara) for 35 cycles (98 °C for 10 s, 60 °C for 15 s, 68 °C for 30 s) with specific primers (Takara).

Western blot

The mouse whole brain was lysed by Cell-LyEX MP (TOYO B-Net, Tokyo, Japan) and separated 40 μg of the protein of the whole brain lysate by electrophoresis on a 5 to 20 % gradient sodium dodecyl sulfate-poly-acrylamide gel (Bio-Rad, Hercules, CA, USA). The proteins were transferred onto a polyvinylidene fluoride membrane. The membrane was subjected to the western blot analysis. After the blocking, the membrane

was incubated with a polyclonal rabbit anti-HNMT antibody (Abcam, Cambridge, UK) and a polyclonal rabbit anti-β-actin antibody (Cell Signaling Technology, Danvers, MA, USA). After washing, the membrane was incubated with peroxidase-linked anti-rabbit IgG (Thermo Fisher Scientific, Waltham, MA, USA) and the blots were developed using ECL reagents (Bio-Rad). The signals were acquired with a luminescent image analyser, Ez-Capture MG (Atto, Tokyo, Japan).

Measurement of HNMT enzymatic activity

HNMT activity was measured by a radiometric method as described previously[12]. In brief, we exchanged the buffer of brain homogenates with 125 mM bicine (pH 8.2) using a gel filtration column, Zeba Spin Desalting Column (Thermo Fisher Scientific). Then, the homogenates were incubated at 37 ºC for 60 min with 0.125 μCi of [^{3}H]-labeled s-adenosylmethionine (SAM) (PerkinElmer, Waltham, MA, USA), 40 μM non-labeled SAM (New England Biolabs, Ipswich, MA, USA), 40 μM histamine (Sigma-Aldrich, St. Louis, MO, USA), 1 mM EDTA and 0.025% BSA (Sigma-Aldrich). The reaction was terminated by adding 150 μL of 1.25 M sodium borate (pH 11.0).

Then, 1.25 mL of toluene/isopentanol (1:1) was added to each tube. After shaking followed by centrifugation at 2,000 × *g* for 5 min, 1 mL of organic layer was transferred to new tubes and mixed with 250 μL of 0.5 N HCl. After shaking followed by centrifugation at 2,000 × *g* for 5 min, 200 μL of aqueous layer was transferred to scintillation vials and mixed with 2 mL of Ultima Gold XR (PerkinElmer). Radioactivity was measured using a liquid scintillation counter, LS-6500 (Beckman Coulter, Brea, CA, USA). In this experiment, 125 mM bicine without homogenates was used as a blank and the count of the blank was subtracted from the total count of each fraction to calculate specific enzymatic activities.

Monoamine neurotransmitters and their metabolites measurement

The brain homogenate samples were prepared by same methods of the histamine and 1-methylhistamine measurements. Sample separation took place at 25 ºC on an SC-5ODS column (2.1 i.d. × 150 mm; EICOM) using a 0.1 M citric acid/ 0.1 M acetic acid (pH 3.9)-methanol (83:17, v/v) buffer containing 140 mg/L sodium 1-octanesulfonate and 5 mg/L EDTA/2Na with a flow rate of 230 μL/min. Monoamine

neurotransmitters (5-HT, DA, and NE) and their metabolites (5-HIAA, DOPAC, 3-MT, and HVA) were measured by electrochemical detector (HTEC-500; EICOM).

Statistical analysis

All results are presented as mean ± S.E.. Data were analysed by two-tailed paired student's *t*-test using the GraphPad Prism version 5 software package (GraphPad, La Jolla, CA, USA). Differences were considered significant at P values less than 0.05.
